# Development of a preprocedure nomogram for predicting contrast-induced acute kidney injury after coronary angiography or percutaneous coronary intervention

**DOI:** 10.18632/oncotarget.20519

**Published:** 2017-08-24

**Authors:** Bao-Liang Guo, Fu-Sheng Ouyang, Shao-Ming Yang, Zi-Wei Liu, Shao-Jia Lin, Wei Meng, Xi-Yi Huang, Li-Zhu Ouyang, Hai-Xiong Chen, Qiu-Gen Hu

**Affiliations:** ^1^ Department of Radiology, Shunde Hospital of Southern Medical University, The First People’s Hospital of Shunde, Foshan, Guangdong, P.R. China; ^2^ Department of Radiology, Lecong Hospital of Shunde, Foshan, Guangdong, P.R. China

**Keywords:** contrast-induced acute kidney injury, nomogram, coronary angiography, percutaneous coronary intervention

## Abstract

Most of the risk models for predicting contrast-induced acute kidney injury (CI-AKI) are available for postcontrast exposure prediction, thus have limited values in practice. We aimed to develop a novel nomogram based on preprocedural features for early prediction of CI-AKI in patients after coronary angiography (CAG) or percutaneous coronary intervention (PCI). A total of 245 patients were retrospectively reviewed from January 2015 to January 2017. Least absolute shrinkage and selection operator (Lasso) regression model was applied to select most strong predictors for CI-AKI. The CI-AKI risk score was calculated for each patient as a linear combination of selected predictors that were weighted by their respective coefficients. The discrimination of nomogram was assessed by C-statistic. The occurrence of CI-AKI was 13.9% (34 out of 245). We identified ten predictors including sex, diabetes mellitus, lactate dehydrogenase level, C-reactive protein, years since drinking, chronic kidney disease (CKD), stage of CKD, stroke, acute myocardial infarction, and systolic blood pressure. The CI-AKI prediction nomogram obtained good discrimination (C-statistic, 0.718, 95%CI: 0.637-0.800, *p* = 7.23 × 10^-5^). The cutoff value of CI-AKI risk score was -1.953. Accordingly, the novel nomogram we developed is a simple and accurate tool for preprocedural prediction of CI-AKI in patients undergoing CAG or PCI.

## INTRODUCTION

Contrast-induced acute kidney injury (CI-AKI) is one of the common complications of coronary diagnostic and interventional procedures, which is significantly associated with prolonged hospitalization, health cost and increased mortality [1, 2]. Although the definition of CI-AKI varies, it is usually defined as an increase in the serum creatinine (Scr) level of 25% or an increase of 0.5 mg/dL (or 44 μmol/L) from baseline within 48-72 h of contrast exposure [3, 4]. Identifying patients at risk of CI-AKI easily and accurately would allow the administration of prophylactic interventions to those at high risk [5, 6]. The effect of CI-AKI on morbidity and mortality will be shaped by advances in methods to detect CI-AKI earlier in the disease course.

Several clinical risk models for prediction of CI-AKI have been identified, which can be roughly classified into post contrast exposure models and pre-procedural or pre-contrast exposure models. The majority of those proposed models were post-contrast models that be used when patients have been exposed to contrast medium, and the accuracy of these models yielded C-statistics ranging from 0.61 to 0.924 [7-16]. Mehran’s risk score model yielding a C-statistics of 0.69, is one of the most influential models, so is the Bartholomew score [15, 17]. However, those post-contrast models may be a little time-insensitive and to develop a risk model that using less easily calculated variables which can achieve quick applicability of CI-AKI risk prior to patients being subjected to contrast exposure may be more clinically attractive. Though Maioli, Chen and Inohara proposed preprocedural scores for predicting the risk of CI-AKI with seven or more risk factors, all of them were a lack of quantitative biomarkers that may serve as important predictors in risk score models [11, 18, 19].

Thus, there is an increasing need for a more objective and simple identification tool which can be readily available for prediction of CI-AKI on admission. The goal of this study is to establish a novel simple and accurate risk model that can early predict CI-AKI before procedure.

## RESULTS

### All patients

A total of 245 consecutive patients (mean age: 65.7 ± 11.0 years) who underwent coronary angiography (mean contrast volume: 122.1 ± 76.3 mL) were included in the final analysis. Table 1 shows the comparsion of baseline characteristics between CI-AKI group and non-CI-AKI group. The results provide little value to the prediction of CI-AKI. Among the 245 patients, 34 (13.9%) patients developed CI-AKI. Of these, 18 (52.9%) patients developed CI-AKI on day 1, 22 (64.7%) patients developed CI-AKI on day 2, and 34 (100%) patients developed CI-AKI on day 3.

**Table 1:** Comparsion of patient characteristics between the CI-AKI group (n = 34) and non-CI-AKI group (n = 211)

Characteristics	CI-AKI group	Non-CI-AKI group	*p*-value
Age (yrs.)	66.1 ± 11.3	65.6 ± 11.0	0.907
Male (n, %)	18 (52.9%)	149 (70.6%)	0.048
Current smoking	14 (41.2%)	85 (40.3%)	1.000
Years since smoking	11.0 ± 15.2	12.5 ± 17.0	0.820
Current drinking	9 (26.5%)	41 (19.4%)	0.362
Years since drinking	7.1 ± 13.1	4.9 ± 11.6	0.322
DM	11 (32.4%)	51 (24.2%)	0.297
Years since DM	2.1 ± 5.0	1.6 ± 3.8	0.481
Hypertension	24 (70.6%)	149 (70.6%)	1.000
Hypertension grading	2.0 ± 1.3	1.8 ± 1.2	0.402
Years since hypertension	6.2 ± 6.6	5.4 ± 6.2	0.507
LDH (U/L)	235.5 ± 78.9	279.1 ± 239.4	0.234
Hs-CRP (mg/L)	6.01 ± 9.8	11.2 ± 25.4	0.034
CKD	0	14 (6.6%)	0.228
Stage of CKD (0-3)	0	0.2 ± 0.6	< 0.001
Stroke	1 (2.9%)	18 (8.5%)	0.486
Acute MI	13 (38.2%)	57 (27.0%)	0.219
Admission SBP (mmHg)	137.4 ± 22.9	133.7 ± 21.6	0.360
Admission DBP (mmHg)	77.6 ± 10.6	78.9 ± 12.8	0.603
NYHA grade	2.1 ± 0.9	2.0 ± 1.0	0.296
Contrast dose (mL)	107.5 ± 49.3	124.4 ± 79.7	0.230
Baseline Scr (mg/dL)	113.1 ± 82.7	109.0 ± 68.3	0.757
GHbA1c (%)	21.4 ± 85.6	6.4 ± 1.6	0.316
Serum sodium (mmol/L)	140.2 ± 3.0	140.0 ± 3.9	0.751
CK (U/L)	138.8 ± 122.0	256.6 ± 765.5	0.372
CK-MB (U/L)	16.3 ± 9.3	20.1 ± 33.0	0.505
GOT (U/L)	28.8 ± 27.3	47.9 ± 175.7	0.527

### Risk score and risk model development

**Figure 1:**
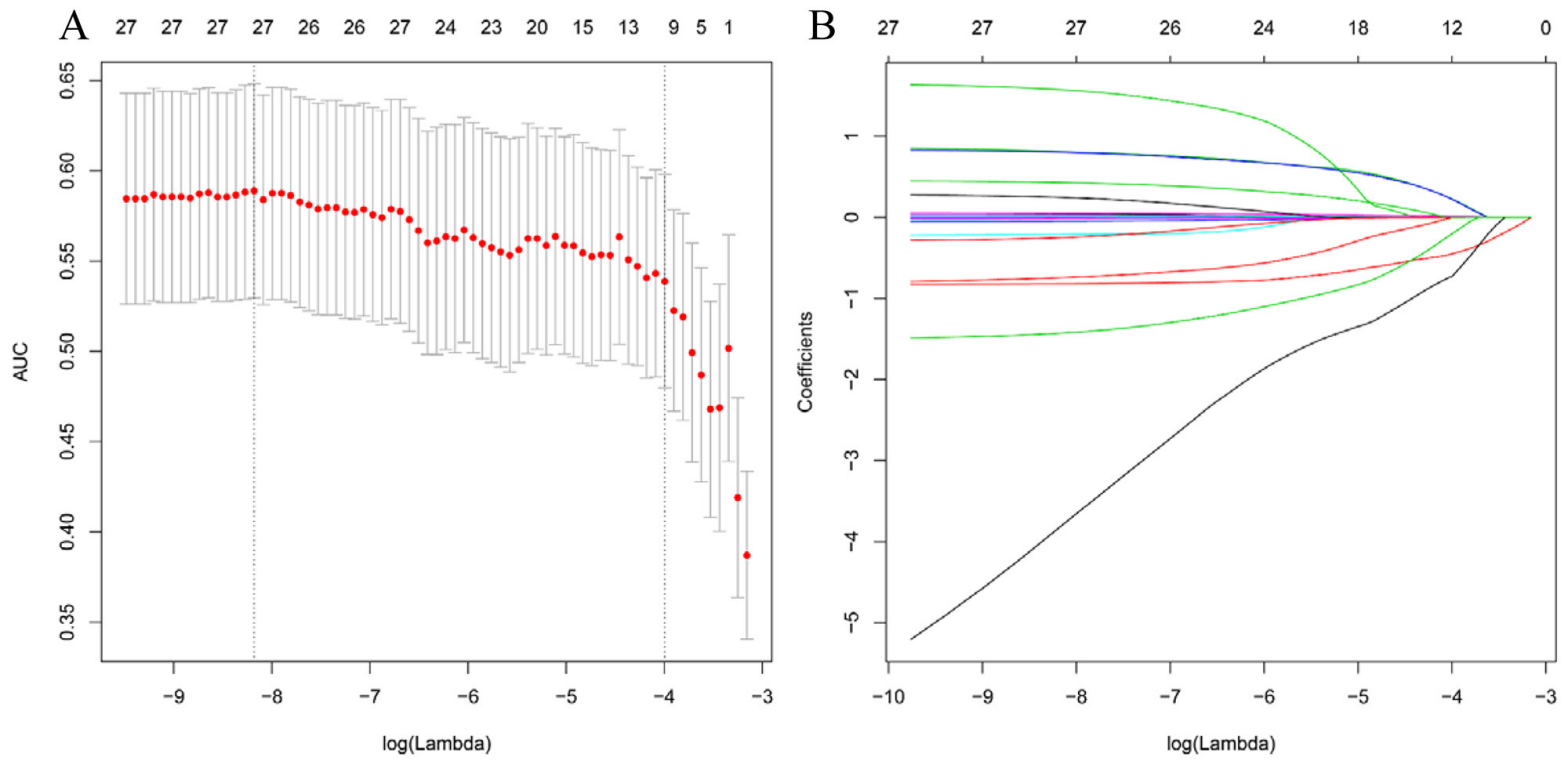
Predictor selection using the least absolute shrinkage and selection operator (LASSO) logistic regression model **(A)** Identification of the optimal penalization coefficient lambda (λ) in the Lasso model used 10-fold cross-validation and the minimum criterion. **(B)** Lasso coefficient profiles of the 26 clinical features. The dotted vertical line was plotted at the value selected using 10-fold cross-validation in Figure A, for which the optimal λ resulted in 10 non-zero coefficients.

After variable selection by lasso regression model, sex, DM, LDH, Hs-CRP, years since drinking, CKD, stage of CKD, stroke, acute MI, and SBP were selected as the best subset of risk factors to develop the CI-AKI risk score and risk model (nomogram) (Figure 1). CI-AKI risk score calculation formula was as follows:

CI-AKI risk score = 0.2232*DM + 0.2188* acute MI + 0.013*years since drinking + 0.0025*SBP – 0.4489*sex – 0.0001*LDH – 0.0011* Hs-CRP – 0.725*CKD – 0.0013* stage of CKD – 0.1994*stroke – 2.0130.

Where, male was labled as 1 and female was labeled as 0; for DM, acute MI, CKD, and stroke, yes was labeled as 1, no was labeled 0; stage of CKD was labeled as 0-3.

**Figure 2:**
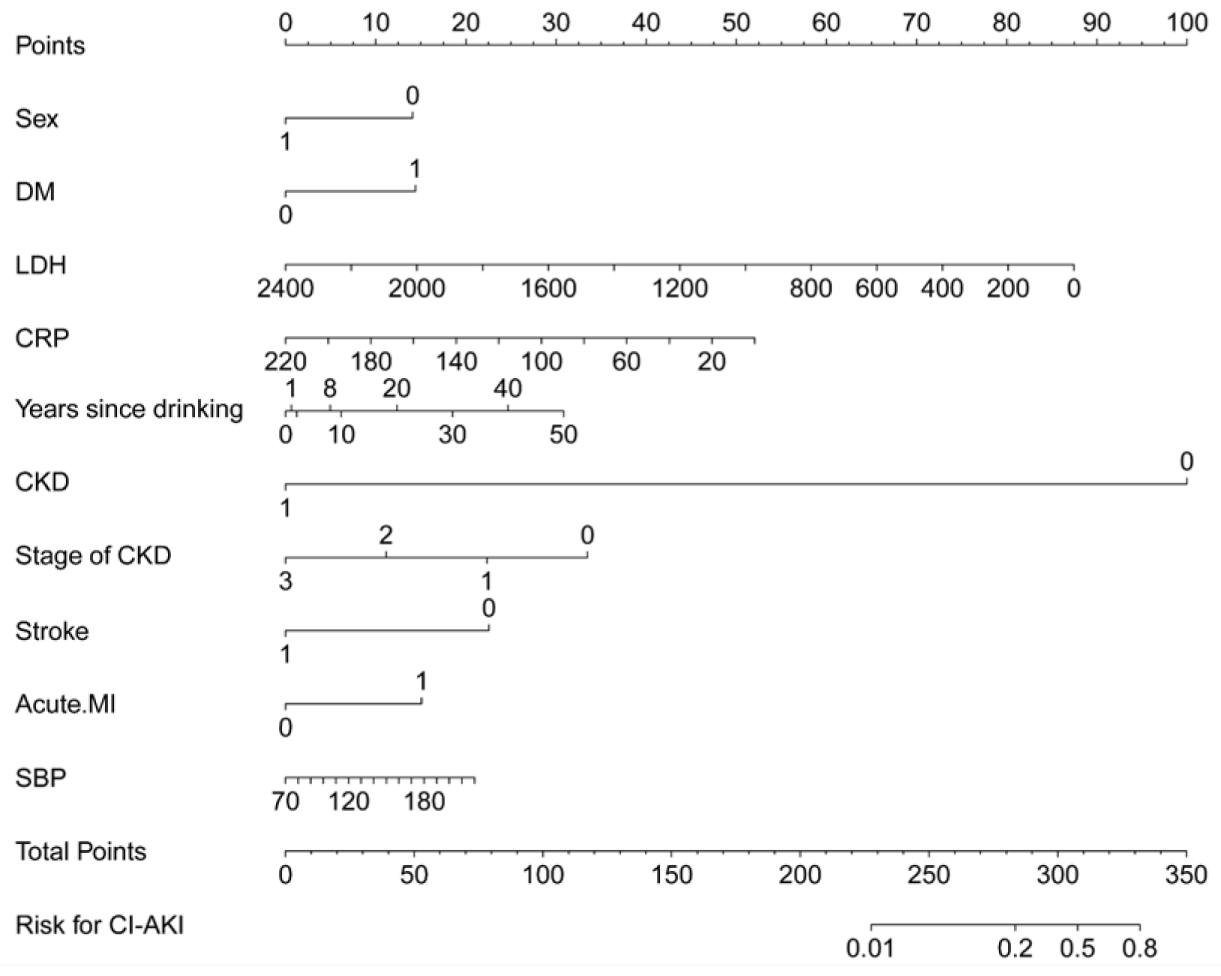
A CI-AKI prediction nomogram integrated the predictors selected by Lasso, including sex, DM, LDH, Hs-CRP, years since drinking, CKD, stage of CKD, stroke, acute MI, and admission SBP The nomogram had excellent discriminative power with a C-statistic of 0.718 (95%CI: 0.637-0.800, *p* = 7.23 × 10-5) and was well calibrated with Hosmer-Lemeshow χ^2^ statistic of 5.829 (*p* = 0.120).

We divided patients into high-risk and low-risk groups according to cutoff value of CI-AKI risk score. The cutoff value of risk score was -1.953. Patients with risk score ≤ -1.953 were classified into high-risk group, while patients with risk score>-1.953 were classified into low-risk group. The nomogram had excellent discriminative power with a C-statistic of 0.718 (95%CI: 0.637-0.800, *p* = 7.23 × 10^-5^) and was well calibrated with Hosmer-Lemeshow χ^2^ statistic of 5.829 (*p* = 0.120) (Figure 2). The predicted probabilities of developing CI-AKI ranging from 0.01% to 80%.

## DISCUSSION

In the present study, for the first time, we developed a novel nomogram for prediction of CI-AKI based on ten readily available predictors (sex, DM, LDH, Hs-CRP, years since drinking, CKD, stage of CKD, stroke, acute MI, and SBP) at pre-procedure. The proposed risk model showed high discrimination and well calibration in our dataset. Because a quantitative risk score allows different potential decision, we provided two possible definitions of low and high-risk groups for CI-AKI for physicians’ choices of cut points that are more or less stringent as needed. This proposed simple risk model allows for immediate identification of high-risk patients before the procedure and appropriate (and timely) risk allocation.

Of note, together with well-known risk factors, the proposed score also includes two biomarkers, LDH and Hs-CRP. This is not entirely surprising since pre-existing DM, CKD, and clinical stage of CKD are clearly associated with increased risk of CI-AKI. Because the incidence of CI-AKI in the general population is low, but increases exponentially in patients with pre-existing renal insufficiency or diabetes mellitus [24]. Elevated LDH level may indicate myocardial infarction in patients with coronary heart disease [25]. Myocardial infarction will reduce the left ventricular ejection fraction. A strong correlation is known to exist between cardiovascular disease and renal disease. Hs-CRP, as a marker of systemic inflammation, has been proven to be associated with an increased risk for CI-AKI in patients undergoing coronary angiography [26]. Previous studies suggested that various inflammatory-related factors were important contributors to the development of CI-AKI and among them, Hs-CRP emerged as a powerful one.

Since the treatment of CI-AKI is rather limited and the prognosis of CI-AKI always ends up with prolonged hospital stay, unfavorable in-hospital and long-term clinical outcomes, a practical and effective solution to this complication is its prevention [20]. So the advances in methods to detect CI-AKI earlier are urgently needed. Before this study, Maioli [18], Chen [19] and Inohara [11] developed pre-procedural scores for risk of contrast-induced nephropathy (CIN) or CI-AKI with seven or more factors yielding a C-statistics of 0.86, 0.82 and 0.80, respectively. We could not compare with these scores because some risk factors were not readily available in our hospital, such as left ventricular ejection fraction ≤ 45%, baseline creatinine clearance ≤ 44 or < 60 mL/min, posthydration creatinine ≥ prehydration creatinine value and one procedure effected within the past 72 h. However, the quantitative biomarkers used in our risk model may be more available than most risk factors used in those three scores. All ten predictors could be easily obtained from our medical records. Furthermore, unlike the other scores, we used powerful machine-learning method lasso regression algorithm to select predictors that most associated with the CI-AKI. We could calculate CI-AKI risk score for each patient and indentified cutoff value of the risk score. Patients with risk score higher than -1.953 were deemed to be high-risk of CI-AKI. We then applied prediction nomogram to generate individual possibility of CI-AKI based on total points of each patient. The nomogram tells us that we should pay close attention to patients with CKD.

Compared to the widely used Mehran’s CIN score (C-statistics = 0.69), our CI-AKI risk score achieves higher predictive power and uses more available pre-procedural risk factors. Timely pre-procedural risk prediction makes many potential benefits in general. It can help the interventional team to establish more individually tailored procedures such as appropriately reduce the dose of contrast volume following with sufficient hydration or combined with pharmacological prophylaxis or to adopt some other precautionary measures on those patients more likely to develop CI-AKI. Patients at high-risk can also choose alternative imaging methods or opt out of the further investigation. It can also allow clinical trials and quality improvement interventions to target patients most prone to benefit from the system based quality improvement efforts provided by Brown and colleagues which reduced the rate of CIN by 20% in consecutive patients with PCI at multiple centers through a multifaceted intervention [27]. Samuel et al. recommended that the clinicians should consider using the scores that do not include contrast volume to estimate a patient’s risk of CIN. Thus our risk score is clinically attractive [28].

Some limitations to this study should be acknowledged. Firstly, retrospective study in nature. Secondly, because this study was conducted in a single center with a relatively small sample size, the risk score model needs to validate and recalibrate further for more widespread use. Thirdly, we couldn’t include more risk factors reported in the previous studies due to they were unavailable.

The generated risk score model in this study is a simple and accurate tool for early prediction of CI-AKI in patients after CAG or PCI at pre-procedure. It can be used for both clinical and investigational purposes. It could help clinicians to assess the risk of CI-AKI before contrast exposure, plan and initiate the most appropriate disease management in time.

## MATERIALS AND METHODS

This reprospectively designed observational study included consecutive patients who underwent coronary angiography (CAG) or percutaneous coronary intervention (PCI) between January 2015 and January 2017. We included patients aged ≥ 18 years who had stayed in the hospital for 2-3 days after coronary angiography. Serum creatinine concentrations was measured in these patients at hospital admission before coronary angiography and on days 1, 2, and 3 after procedure. The exclusions were identified according to the updated European Society of Urogenital Radiology Contrast Media Safety Committee guidelines [5]. The institutional Ethics Research Committee approved the study, and the written informed consent was waived from all patients

### Coronary angiography

Coronary angiography was performed according to standard clinical practice, using standard guide catheters, guidewires, balloon catheters, and stents via the femoral or radial approach. The volume of contrast was at the discretion of the interventional cardiologist. All patients received nonionic, low-osmolarity contrast medium (Ultravist, 370 mg I/mL, Bayer Healthcare). Subjects were treated according to AHA/ACCF guidelines [20]. According to the local institutional protocol, serum creatinine level was measured at hospital admission and on days 1, 2, and 3 after coronary angiography.

### Outcome definition

The clinical endpoint was CI-AKI, defined as an absolute increase in serum creatinine of ≥ 0.5 mg/dL (or 44 μmol/L) or a relative SCr increase ≥ 25% from baseline within 72 hours after contrast medium exposure.

### Candidate predictors

Candidate predictors included clinical variables such as age, sex, diabetes mellitus (DM), years since DM, pre-existing hypertension, lactate dehydrogenase (LDH), high-sensitivity C-reactive protein (Hs-CRP), current drinking, years since drinking, current smoking, years since smoking, pre-existing chronic kidney disease (CKD) defined as pre-admission CrCl (calculated by Cockcroft–Gault formula) < 60 ml/min/1.73m^2^, stage of CKD (stages 0-3), history of stroke, acute myocardial infarction (MI), systolic blood pressure (SBP), diastolic blood pressure (DBP), hypertension grading, NYHA grade, contrast dose, baseline serum creatinine concentrations (Scr), glycosylated hemoglobin (GHbA1c), serum sodium, creatine kinase (CK), creatine kinase MB (CK-MB), glutamic-oxalacetic transaminease (GOT). Of these, sex, DM, current drinking, current smoking, pre-existing CKD, stage of CKD, stroke, acute MI were categorical variables. The remaining variables were continuous variables. All these variables were assessed and recorded on admission. The baseline serum creatinine measured before procedure were used to develop the nomogram for early prediction of CI-AKI, and those in 48 h and 72 h after the procedure were used to evaluate the development of CI-AKI.

### Predictor selection

We used least absolute shrinkage and selection operator (Lasso) method to select features that were most significant and then built a regression model including selected variates [21]. Originally proposed for the linear regression model, this method minimizes the residual sum of squares, subject to the sum of the absolute value of the coefficients being less than a tuning parameter (λ). For the binary logistic regression model, the residual sum of squares is replaced by the negative log-likelihood. If the λ is large, there is no effect on the estimated regression parameters, but as the λ gets smaller, some coefficients may be shrunk towards zero [22, 23]. We then selected the λ for which the cross-validation error is the smallest. Finally, the model is re-fit using all of the available observations and the selected λ. By the way, most of the coefficients of the covariates are reduced to zero and the remaining non-zero coefficients are selected by Lasso. Non-zero coefficient of the selected feature is defined as CI-AKI risk score. The CI-AKI risk score was calculated for each patient as a linear combination of selected features that were weighted by their respective coefficients.

### Statistical analysis

Subsequent analysis was performed using R version 3.2.3 (R Foundation for Statistical Computing). All clinical features (n = 25) were normalized by transforming the data into new scores with a mean of 0 and a standard deviation of 1 (z-score transformation). The package ‘glmnet’ was used for Lasso logistic regression model. The CI-AKI risk score was calculated for each patient as a linear combination of selected predictors that were weighted by their respective coefficients. The ‘rms’ package was used for CI-AKI prediction nomogram. The predictive accuracy of the risk model was assessed by discrimination measured by C-statistic and calibration evaluated by Hosmer-Lemeshow χ^2^ statistic. The differences in various variables between CI-AKI group and non-CI-AKI group were assessed by using an independent samples t test, Chi-square test, or Mann-Whitney U test, where appropriate. All statistical tests were two-sided, and p-values of < 0.05 were considered significant.

## Abbreviations

CI-AKI: Contrast-induced acute kidney injury
CIN: Contrast-induced nephropathy
CAG: Coronary angiography
PCI: Percutaneous coronary intervention
Lasso: Least absolute shrinkage and selection operator
CKD: Chronic kidney disease
Scr: Serum creatinine
DM: Diabetes mellitus
LDH: Lactate dehydrogenase
Hs-CRP: High-sensitivity C-reactive protein
MI: Myocardial infarction
SBP: Systolic blood pressure
DBP: Diastolic blood pressure
GHbA1c: Glycosylated hemoglobin
CK: Creatine kinase
CK-MB: creatine kinase MB
GOT: Glutamic-oxalacetic transaminease
